# Supplementary material for: Osteocytic oxygen sensing controls bone mass through epigenetic regulation of sclerostin
Source: Nat Commun. 2018 Jul 2;9:2557. doi: 10.1038/s41467-018-04679-7 (PMC6028485; doi:10.1038/s41467-018-04679-7)
Supplement: Supplementary file 1 — Supplementary Information [file 41467_2018_4679_MOESM1_ESM.pdf]

Osteocytic oxygen sensing controls bone mass through epigenetic regulation of sclerostin

by Stegen, S. *et al*.

Supplementary Information contains:

Supplementary Figures 1-14

Supplementary Tables 1-4

Supplementary Figure 1

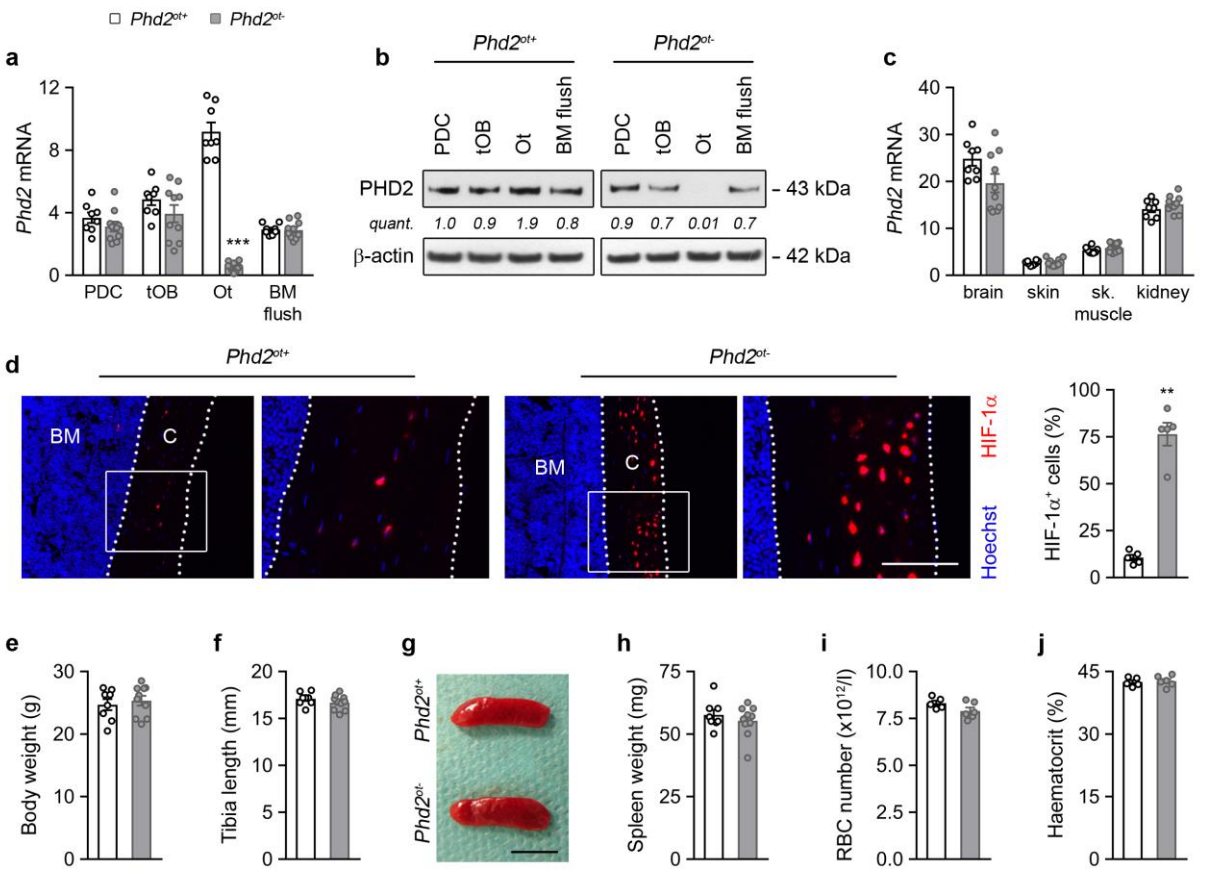

Supplementary Figure 1. Phenotype of *Phd2^{ot-}* mice

(**a**) *Phd2* mRNA levels in periosteum-derived cells (PDC), trabecular osteoblasts (tOB), osteocyte-enriched bone fractions (Ot) or whole bone marrow (BM flush) of 8-week-old mice (n=8 *Phd2^{ot+}* -10 *Phd2^{ot-}*). (**b**) PHD2 and β-actin immunoblot on whole-cell extracts derived from periosteum-derived cells (PDC), trabecular osteoblasts (tOB), osteocyte-enriched bone fractions (Ot) or whole bone marrow (BM flush). Results are representative of three experiments. (**c**) *Phd2* mRNA levels in brain, skin, skeletal (sk.) muscle or kidney (n=4). (**d**) HIF-1α immunostaining of the metaphyseal cortex of tibiae with quantification of the number of HIF-1α-positive osteocytes (n=5). BM is bone marrow, C is cortical bone. Scale bar is 100 µm. (**e**) Body weight of 8-week-old mice (n=8 *Phd2^{ot+}* - 10 *Phd2^{ot-}*). (**f**) Tibia length of 8-week-old mice (n=8 *Phd2^{ot+}* - 10 *Phd2^{ot-}*). (**g-h**) Representative image of spleens of 8-week-old mice (**g**), with quantification (**h**) of spleen weight (n=8 *Phd2^{ot+}* - 10 *Phd2^{ot-}*). Scale bar in (**g**) is 500

µm. (**i-j**) Number of red blood cells (RBCs; **i**) and haematocrit levels (**j**) in peripheral blood of 8-week-old mice (n=5 *Phd2*$^{ot+}$ - 6 *Phd2*$^{ot-}$). Data are means ± SEM. **p<0.01 vs. *Phd2*$^{ot+}$, ***p<0.001 vs. *Phd2*$^{ot+}$ (Student's *t*-test).

Supplementary Figure 2

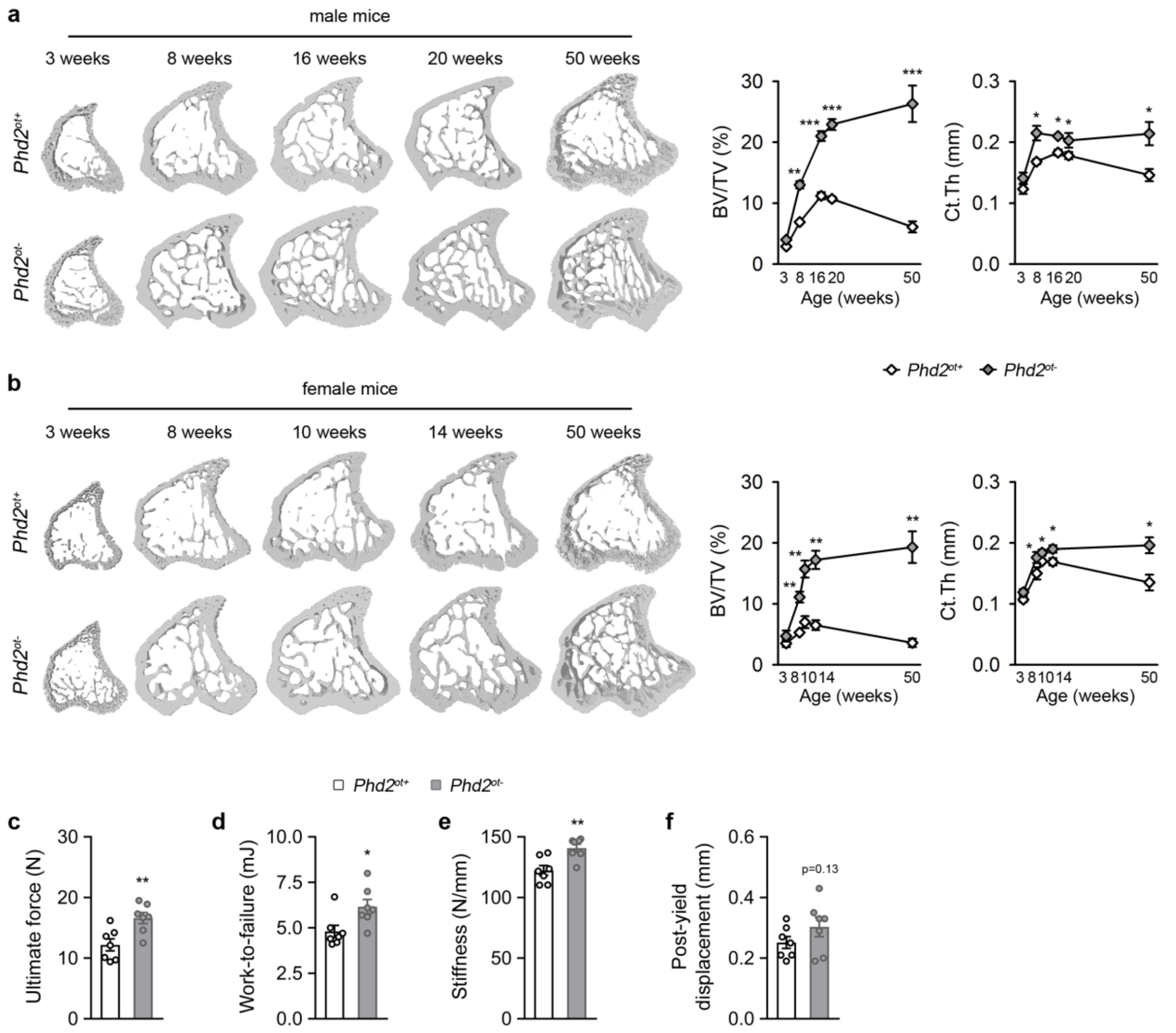

Supplementary Figure 2. *Phd2^{ot-}* mice display increased bone mass and strength

(**a-b**) 3D microCT models of the tibia metaphysis, and quantification of trabecular bone volume and cortical thickness in male (**a**) and female (**b**) mice (n=4). (**c-f**) Biomechanical properties of femora from 8-week-old male mice, as determined by three-point bending analysis (n=7). Data are means ± SEM. *p<0.05 vs. *Phd2^{ot+}*, **p<0.01 vs. *Phd2^{ot+}*, ***p<0.001 vs. *Phd2^{ot+}* (Student's *t*-test).

Supplementary Figure 3

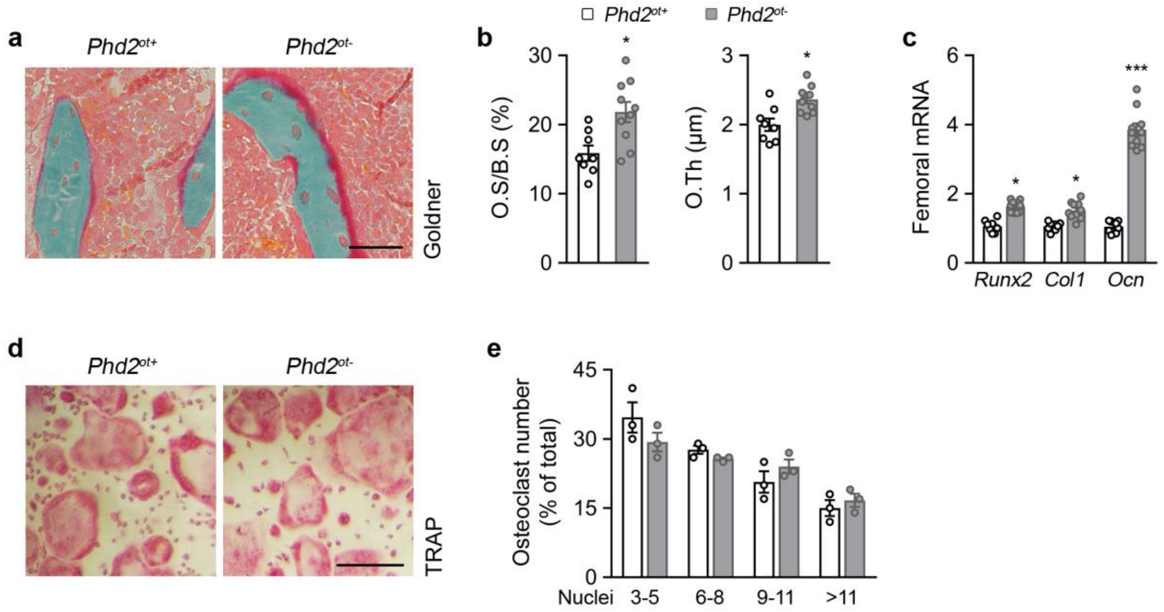

Supplementary Figure 3. Deletion of PHD2 in osteocytes increases osteoid abundance

(**a-b**) Goldner staining (**a**) of the tibial metaphysis with quantification (**b**) of the osteoid surface per bone surface (O.S/B.S) and osteoid thickness (O.Th) in 8-week-old mice (n=8 *Phd2*$^{ot+}$ - 10 *Phd2*$^{ot-}$). (**c**) *Runx2*, *Col1* and *Ocn* mRNA levels in femora of 8-week-old mice (n=8 *Phd2*$^{ot+}$ - 10 *Phd2*$^{ot-}$). (**d-e**) Representative images (**d**) of TRAP-positive multinuclear cells formed after one week of culture with quantification (**e**) of the number of osteoclasts formed per well as percentage of the total number. Quantification was based on the number of nuclei per osteoclast (n=3). Data are means ± SEM. *p<0.05 vs. *Phd2*$^{ot+}$, **p<0.01 vs. *Phd2*$^{ot+}$, ***p<0.001 vs. *Phd2*$^{ot+}$ (Student's *t*-test). Scale bars in (**a**) and (**d**) are 50 µm.

Supplementary Figure 4

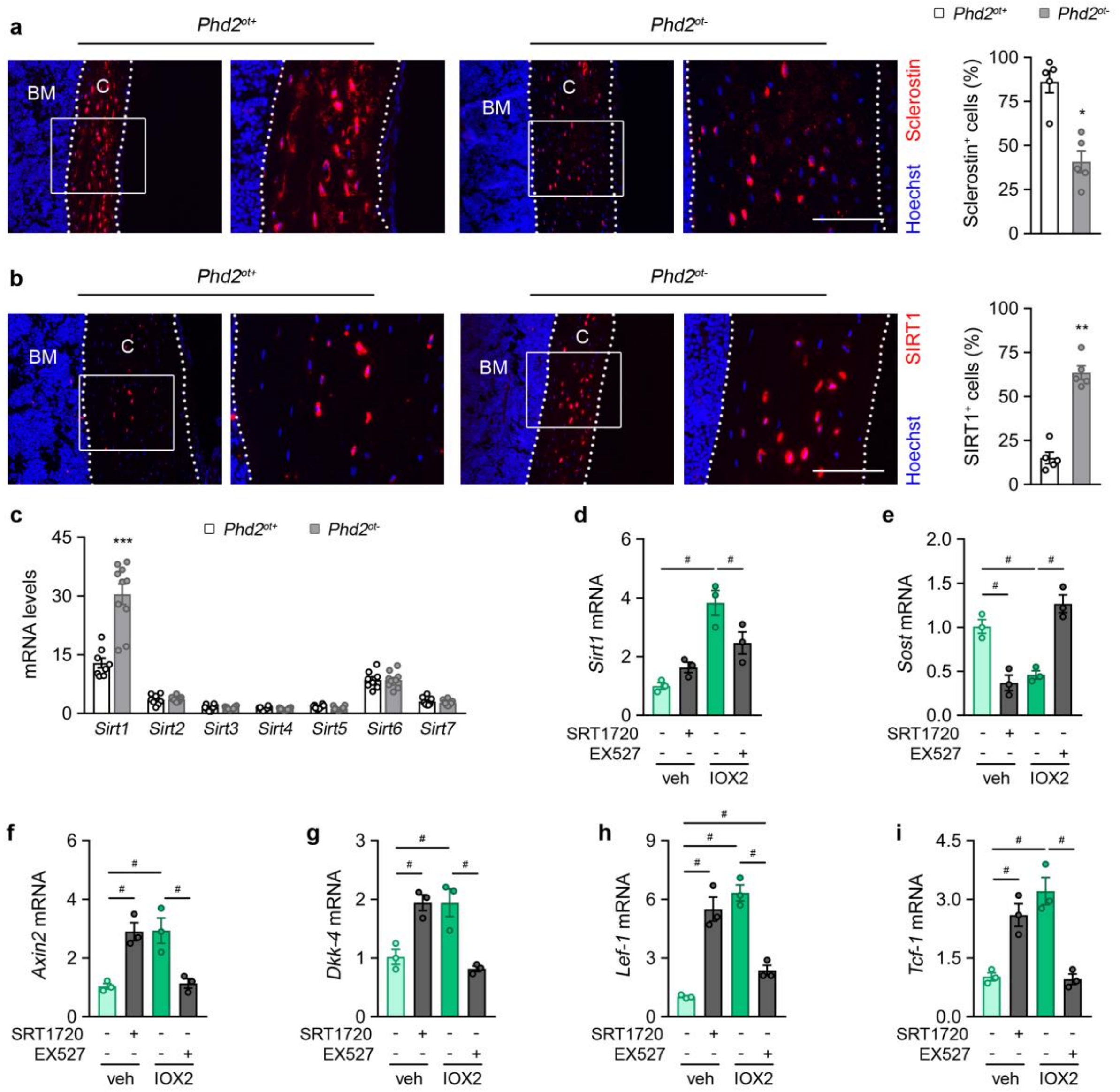

Supplementary Figure 4. Link between PHD2 and SIRT1-sclerostin-WNT signalling

(**a-b**) Sclerostin (**a**) and SIRT1 (**b**) immunostaining of the metaphyseal cortex of tibiae with quantification of the number of sclerostin and SIRT1-positive osteocytes (n=5). BM is bone marrow, C is cortical bone. Scale bars are 100 µm. (**c**) *Sirt1-Sirt7* mRNA levels in osteocyte-enriched bone fractions of 8-week-old mice (n=8 *Phd2*$^{ot+}$ - 10 *Phd2*$^{ot-}$). (**d-i**) *Sirt1* (**d**), *Sost* (**e**), *Axin2* (**f**), *Dkk-4* (**g**), *Lef-1* (**h**) and *Tcf-1* (**i**) mRNA levels in vehicle (IDG^{VEH}) or IOX2-treated IDG-SW3 (IDG^{IOX2}) cells after 14 days of osteogenic differentiation, with or without the addition of SRT1720 or EX527 (n=3). Data are means ± SEM. *$p<0.05$ vs. *Phd2*$^{ot+}$, **$p<0.01$ vs. *Phd2*$^{ot+}$, ***$p<0.001$ vs. *Phd2*$^{ot+}$ (Student's *t*-test), $^{\#}p<0.05$ (two-way ANOVA).

Supplementary Figure 5

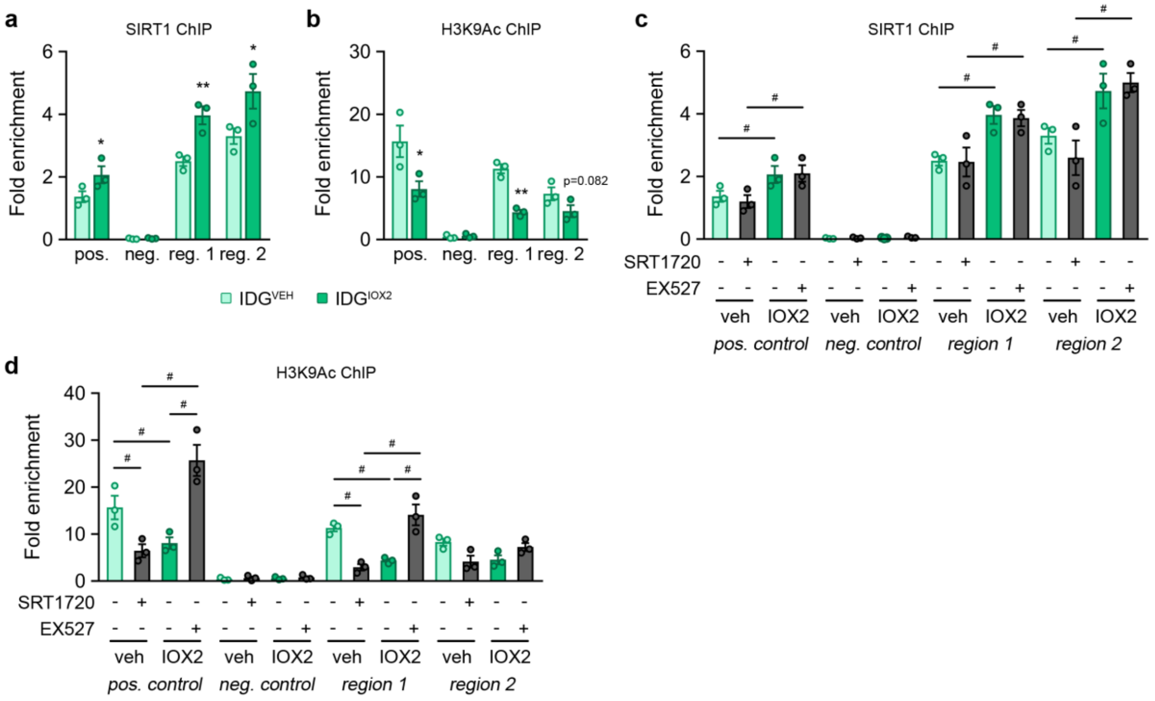

Supplementary Figure 5. SIRT1 binds and deacetylates the *Sost* promoter

(**a-b**) ChIP-qPCR analysis for SIRT1 (**a**) and H3K9Ac (**b**) on specific *Sost* promoter regions in IDG^{VEH} or IDG^{IOX2} cells after 14 days of osteogenic differentiation. Results are fold enrichment compared to input (n=3). (**c-d**) ChIP-qPCR analysis for SIRT1 (**c**) and H3K9Ac (**d**) on specific *Sost* promoter regions in IDG^{VEH} or IDG^{IOX2} cells after 14 days of osteogenic differentiation, with or without the addition of SRT1720 or EX527. Results are fold enrichment compared to input (n=3). Data are means ± SEM. *$p<0.05$ vs. *Phd2*$^{ot+}$, **$p<0.01$ vs. *Phd2*$^{ot+}$ (Student's *t*-test), $^{\#}p<0.05$ (two-way ANOVA).

Supplementary Figure 6

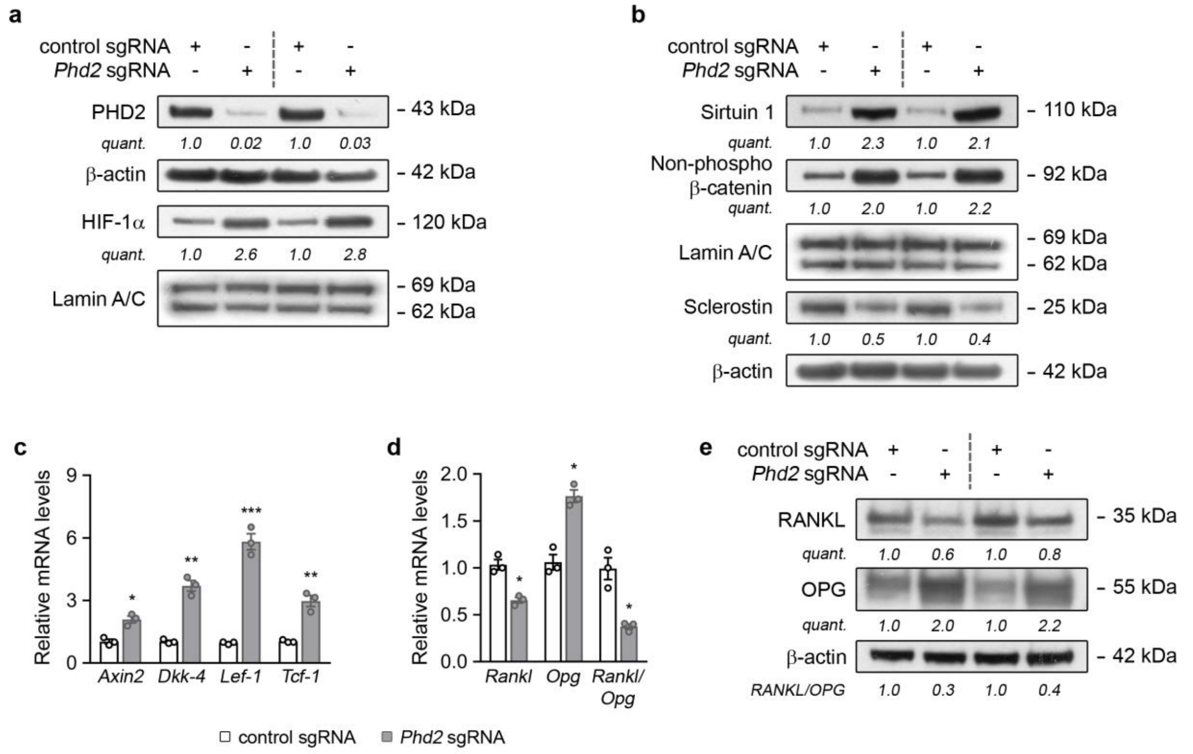

Supplementary Figure 6. Genetic silencing of *Phd2* in IDG-SW3 cells

(**a**) PHD2, β-actin (whole-cell extracts), and HIF-1α and Lamin A/C (nuclear cell extracts) immunoblot. Protein extracts are derived from IDG-SW3 cells after CRISPR-Cas9-mediated deletion of *Phd2*. A scrambled sgRNA was used as control. Results are representative of three experiments. (**b**) Sirtuin 1, non-phosphorylated (non-phospho) β-catenin, Lamin A/C (nuclear cell extracts), and sclerostin and β-actin (whole-cell extracts) immunoblot. Protein extracts were prepared as in (**a**). Results are representative of three experiments. (**c**) *Axin2*, *Dkk-4*, *Lef-1*, *Tcf-1* mRNA levels in IDG-SW3 cells after CRISPR-Cas9-mediated deletion of *Phd2* (n=3). (**d**) *Rankl* and *Opg* mRNA levels, and the *Rankl*/*Opg* mRNA ratio in IDG-SW3 cells after CRISPR-Cas9-mediated deletion of *Phd2* (n=3). (**e**) RANKL, OPG and β-actin immunoblot on IDG-SW3 whole-cell extracts, as prepared in (**a**). Results are representative of three experiments. *p<0.05 vs. control sgRNA, **p<0.01 vs. control sgRNA, ***p<0.001 vs. control sgRNA (Student's *t*-test).

Supplementary Figure 7

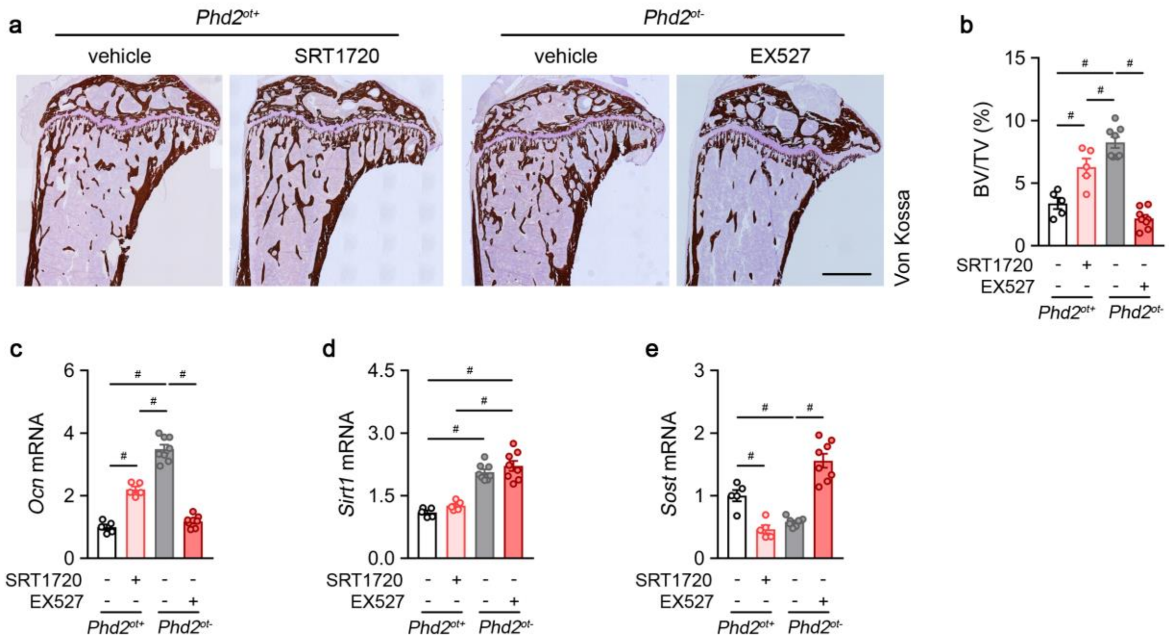

Supplementary Figure 7. SIRT1 regulates bone mass in *Phd2*$^{ot-}$ mice

(**a-b**) Von Kossa staining (**a**) of tibiae with quantification (**b**) of trabecular bone volume (BV/TV) (n=5 *Phd2*$^{ot+}$ - 8 *Phd2*$^{ot-}$). Mice were treated with vehicle, SRT1720 or EX527 for 5 weeks. Scale bar is 500 µm. (**c-e**) *Ocn* (**c**), *Sirt1* (**d**) and *Sost* (**e**) mRNA levels in osteocyte-enriched bone fractions (n=5 *Phd2*$^{ot+}$ - 8 *Phd2*$^{ot-}$). Data are means ± SEM. $^{\#}p<0.05$ (two-way ANOVA).

Supplementary Figure 8

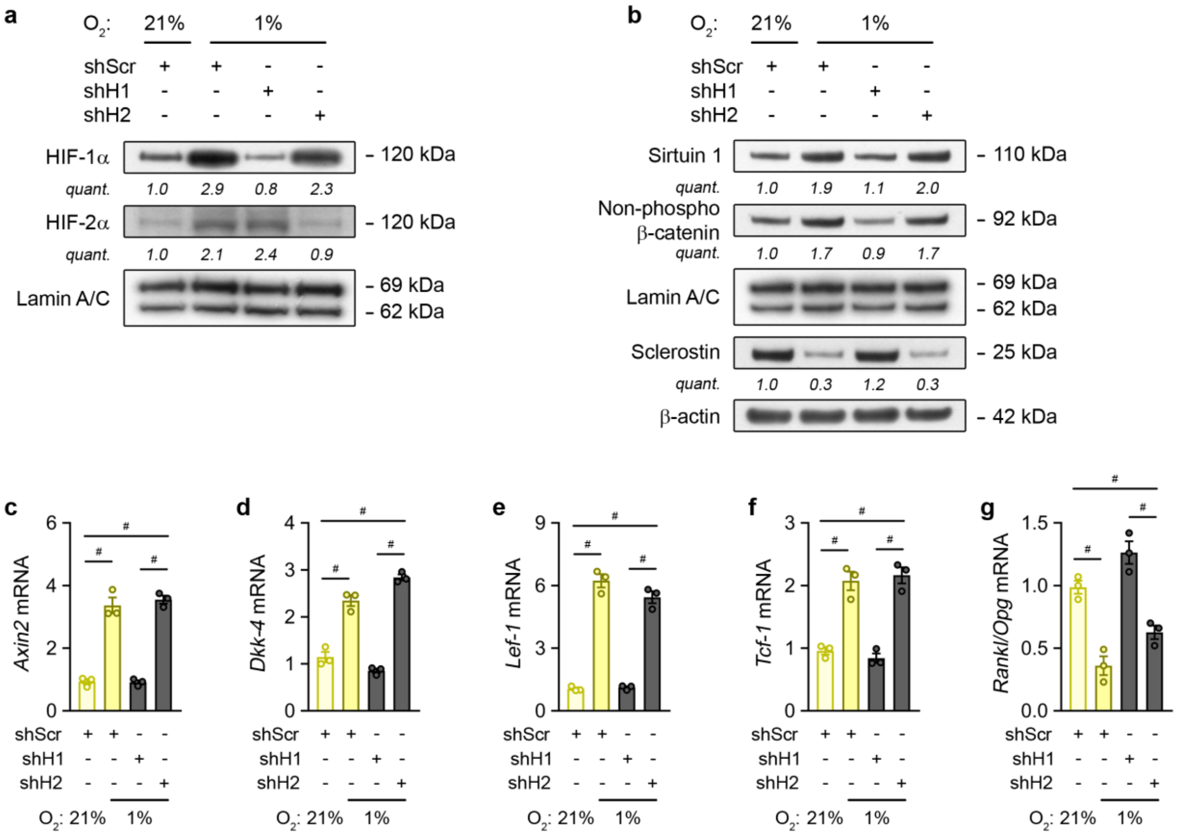

Supplementary Figure 8. Hypoxia enhances SIRT1 levels through HIF-1α

(**a**) HIF-1α, HIF-2α and Lamin A/C immunoblot on nuclear cell extracts derived from IDG-SW3 cells cultured in normoxia (21% O_2) or hypoxia (1% O_2), after transduction with scrambled shRNA (shScr), shHIF-1α (shH1) or shHIF-2α (shH2). Results are representative of three experiments. (**b**) Sirtuin 1, non-phosphorylated (non-phospho) β-catenin, Lamin A/C (nuclear cell extracts), and sclerostin and β-actin immunoblot (whole-cell extracts). Protein extracts are derived from IDG-SW3 cells, cultured in normoxia or hypoxia, after genetic silencing of HIF-1α (shH1) or HIF-2α (shH2). Results are representative of three experiments. (**c-f**) *Axin2* (**c**), *Dkk-4* (**d**), *Lef-1* (**e**), *Tcf-1* (**f**) mRNA levels in IDG-SW3 cells, cultured in normoxia or hypoxia, after genetic silencing of HIF-1α (shH1) or HIF-2α (shH2) (n=3). (**g**) *Rankl* and *Opg* mRNA levels, and the *Rankl*/*Opg* mRNA ratio in IDG-SW3 cells, cultured in normoxia or hypoxia, after genetic silencing of HIF-1α (shH1) or HIF-2α (shH2) (n=3). Data are means ± SEM. $^{\#}p<0.05$ (one-way ANOVA).

Supplementary Figure 9

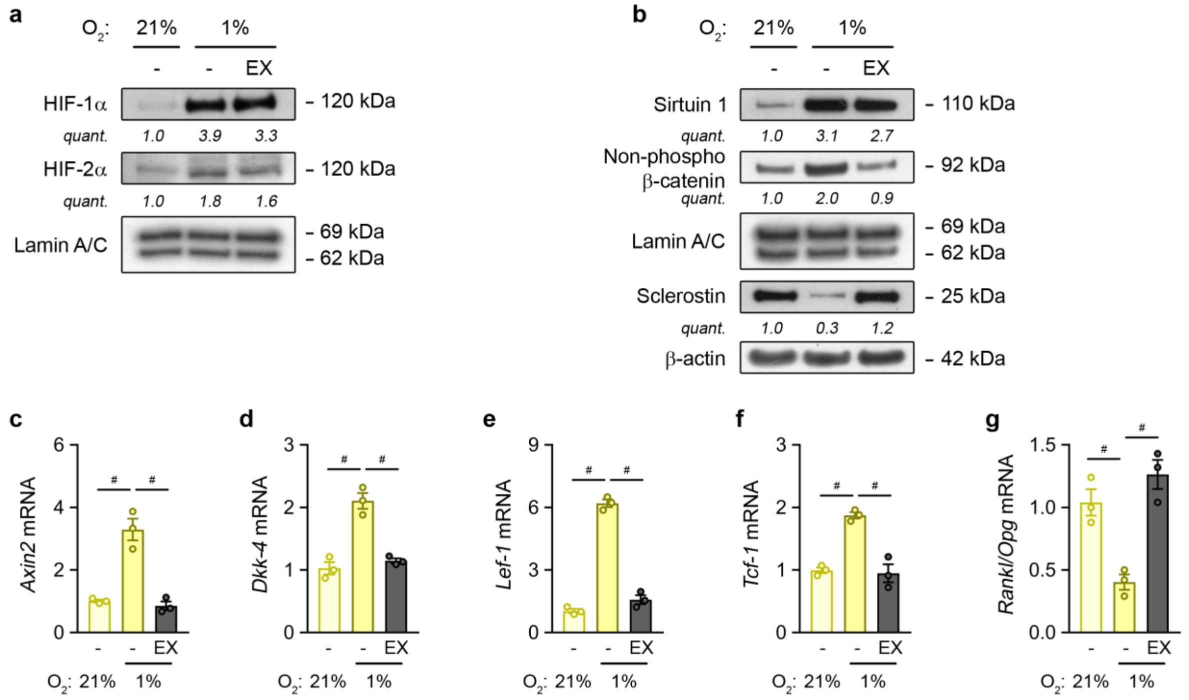

Supplementary Figure 9. SIRT1 controls sclerostin levels in hypoxia

(**a**) HIF-1α, HIF-2α and Lamin A/C immunoblot on nuclear cell extracts derived from IDG-SW3 cells cultured in normoxia (21% O_2) or hypoxia (1% O_2), with or without EX527 (EX) treatment. Results are representative of three experiments. (**b**) Sirtuin 1, non-phosphorylated (non-phospho) β-catenin, Lamin A/C (nuclear cell extracts), and sclerostin and β-actin immunoblot (whole-cell extracts). Protein extracts are derived from IDG-SW3 cells, cultured in normoxia or hypoxia, with or without EX527 treatment. Results are representative of three experiments. (**c-f**) *Axin2* (**c**), *Dkk-4* (**d**), *Lef-1* (**e**), *Tcf-1* (**f**) mRNA levels in IDG-SW3 cells, cultured in normoxia or hypoxia, with or without EX527 treatment (n=3). (**g**) *Rankl* and *Opg* mRNA levels, and the *Rankl*/*Opg* mRNA ratio in IDG-SW3 cells, cultured in normoxia or hypoxia, with or without EX527 treatment (n=3). Data are means ± SEM. $^{\#}p<0.05$ (one-way ANOVA).

Supplementary Figure 10

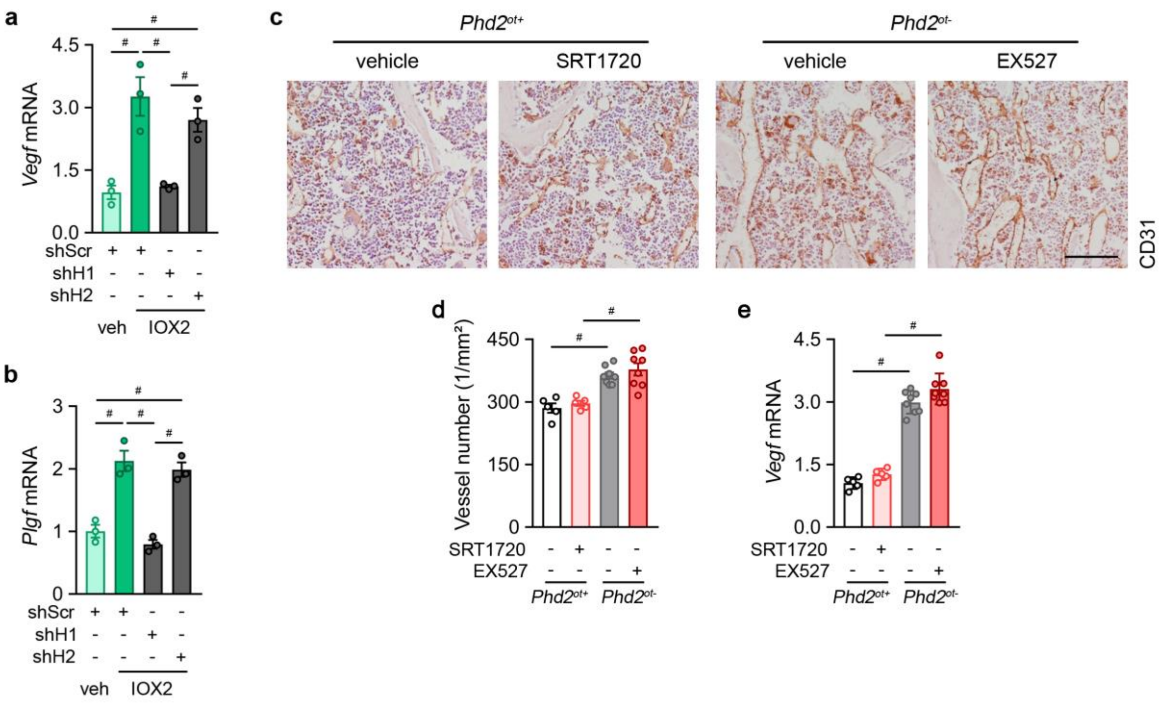

Supplementary Figure 10. Inhibition of PHD2 results in an angiogenic response

(**a-b**) *Vegf* (**a**) and *Plgf* (**b**) mRNA levels in vehicle or IOX2-treated IDG-SW3 cells after genetic silencing of HIF-1α (shH1) or HIF-2α (shH2). A scrambled shRNA (shScr) was used as control (n=3). (**c-d**) CD31 immunostaining (**c**) of the tibial metaphysis with quantification (**d**) of blood vessel number (n=5 *Phd2*$^{ot+}$ - 8 *Phd2*$^{ot-}$). Mice were treated with vehicle, SRT1720 or EX527 for 5 weeks. Scale bar is 100 µm. (**e**) *Vegf* mRNA levels in femora (n=5 *Phd2*$^{ot+}$ - 8 *Phd2*$^{ot-}$). Data are means ± SEM. $^{\#}p<0.05$ (ANOVA: one-way ANOVA in **a**,**b**, two-way ANOVA in **d**,**e**).

Supplementary Figure 11

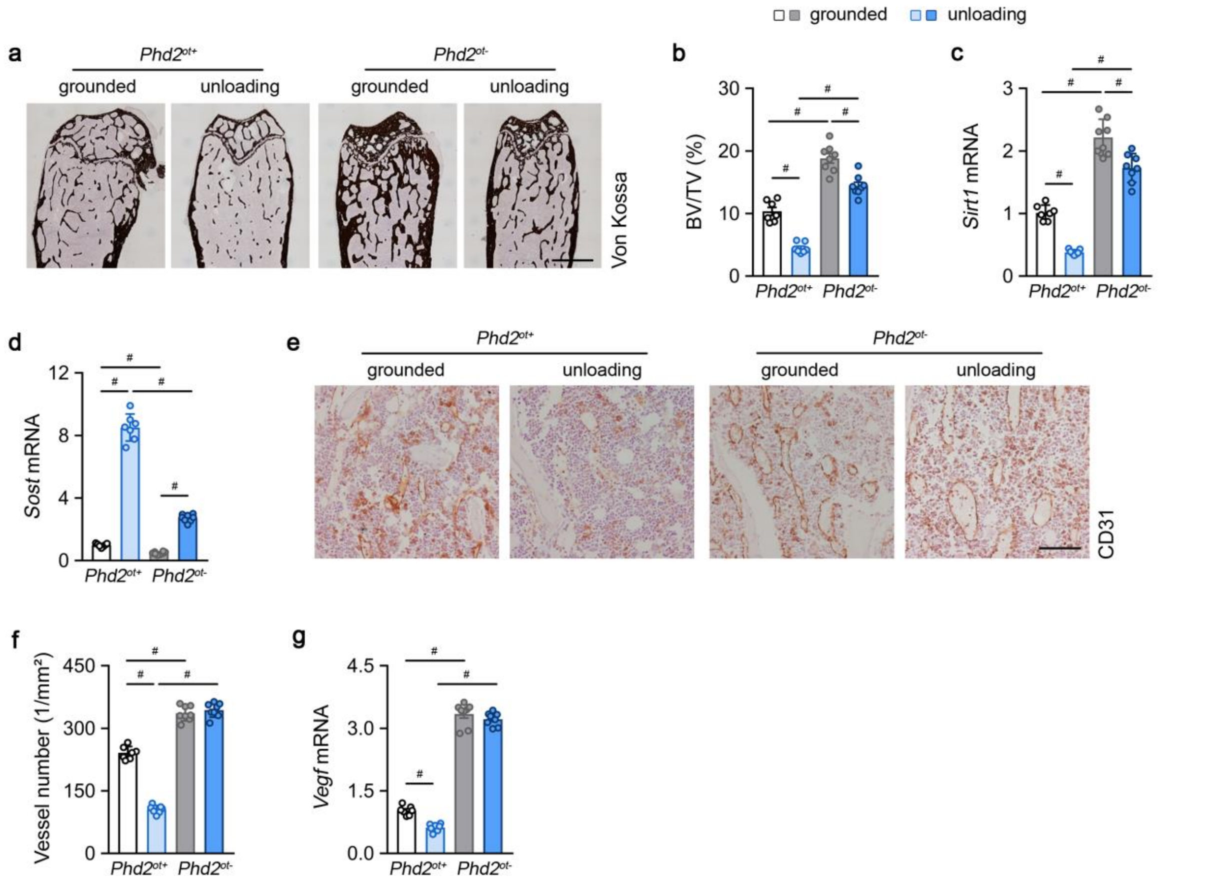

Supplementary Figure 11. *Phd2^{ot-}* mice are protected from disuse-induced bone loss

(**a-b**) Von Kossa staining (**a**) of femora with quantification (**b**) of trabecular bone volume (BV/TV), 4 weeks after hindlimb unloading (n=7 *Phd2^{ot+}* - 8 *Phd2^{ot-}*). (**c-d**) *Sirt1* (**c**) and *Sost* (**d**) mRNA levels in osteocyte-enriched bone fractions (n=7 *Phd2^{ot+}* - 8 *Phd2^{ot-}*). (**e-f**) CD31 immunostaining (**e**) of the tibial metaphysis with quantification (**f**) of blood vessel number (n=7 *Phd2^{ot+}* - 8 *Phd2^{ot-}*). (**g**) *Vegf* mRNA levels in femora (n=7 *Phd2^{ot+}* - 8 *Phd2^{ot-}*). Data are means ± SEM. $^{\#}p<0.05$ (two-way ANOVA). Scale bar in (**a**) is 500 µm, scale bar in (**e**) is 100 µm.

Supplementary Figure 12

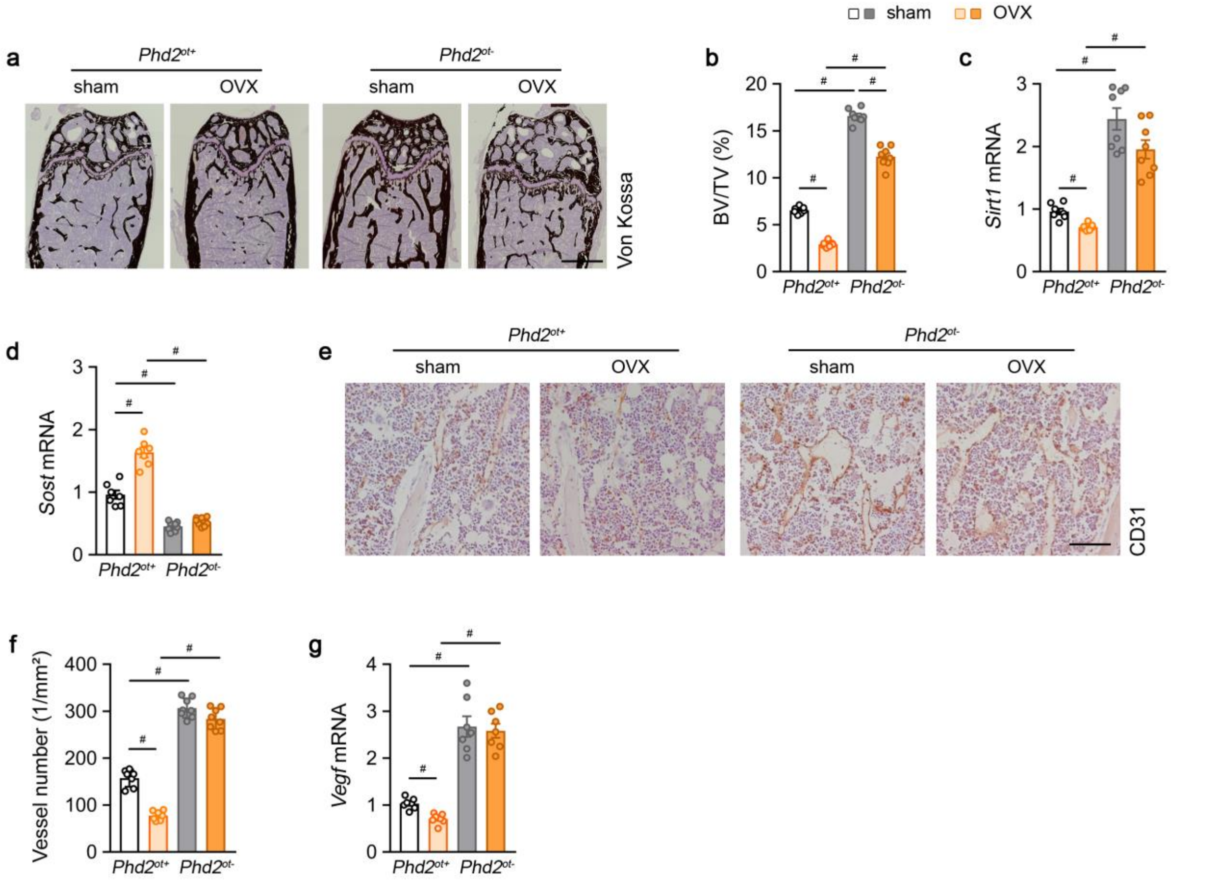

Supplementary Figure 12. *Phd2^{ot-}* mice are protected from OVX-induced bone loss

(**a-b**) Von Kossa staining (**a**) of femora with quantification (**b**) of trabecular bone volume (BV/TV), 4 weeks after OVX (n=7 *Phd2^{ot+}* - 8 *Phd2^{ot-}*). (**c-d**) *Sirt1* (**c**) and *Sost* (**d**) mRNA levels in osteocyte-enriched bone fractions (n=7 *Phd2^{ot+}* - 8 *Phd2^{ot-}*). (**e-f**) CD31 immunostaining (**e**) of the tibial metaphysis with quantification (**f**) of blood vessel number (n=7 *Phd2^{ot+}* - 8 *Phd2^{ot-}*). (**g**) *Vegf* mRNA levels in femora (n=7 *Phd2^{ot+}* - 8 *Phd2^{ot-}*). Data are means ± SEM. $^{\#}p<0.05$ (two-way ANOVA). Scale bar in (**a**) is 500 µm, scale bar in (**e**) is 100 µm.

Supplementary Figure 13

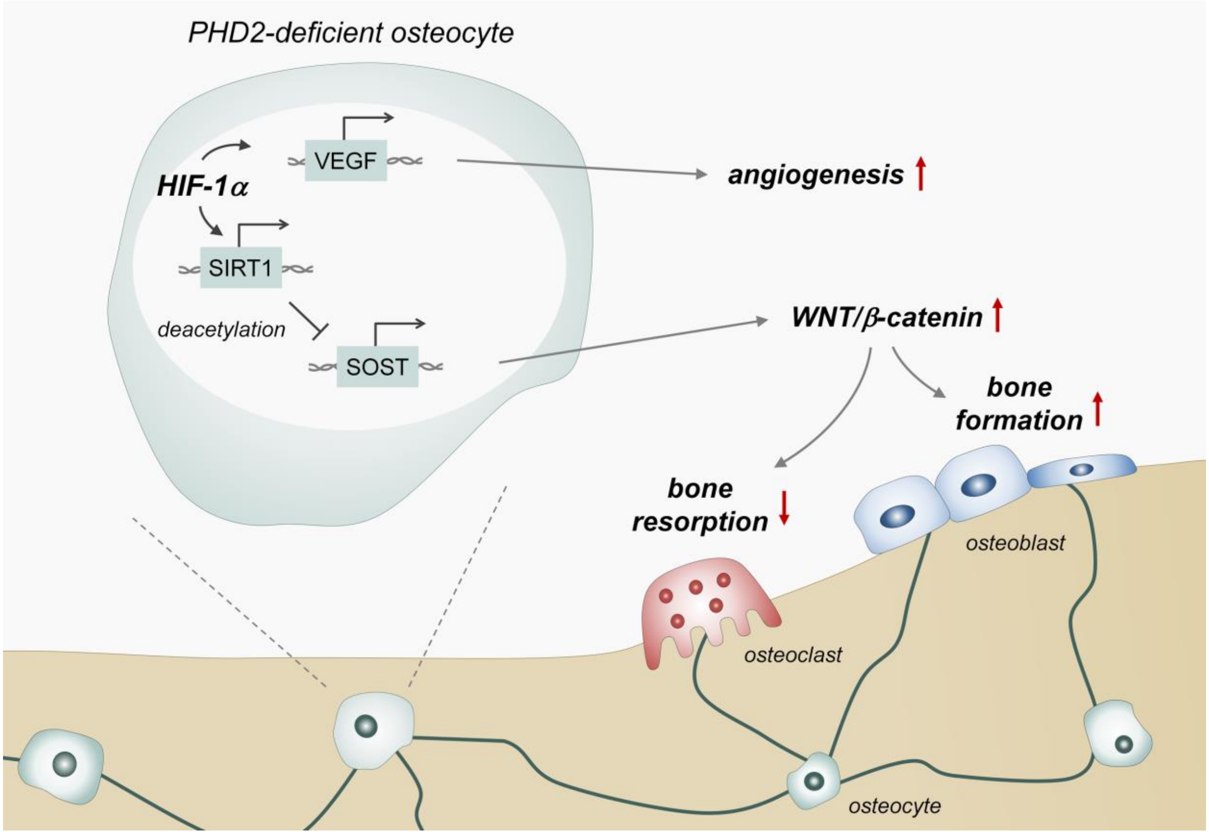

Supplementary Figure 13. Osteocytic PHD2 regulates postnatal bone mass

Deletion of PHD2 in osteocytes results in accumulation of HIF-1α, thereby increasing the expression of Sirtuin 1 (SIRT1), which negatively regulates the expression of sclerostin (SOST) through deacetylation. Downregulation of SOST in PHD2-deficient osteocytes subsequently activates WNT/β-catenin signalling, which leads to enhanced bone formation and decreased bone resorption, ultimately resulting in accumulation of bone mass. This enhanced osteogenic response was accompanied by increased angiogenesis, a process mediated by HIF-1α-dependent production of angiogenic growth factors such as vascular endothelial growth factor (VEGF).

Supplementary Figure 14. Uncropped scans

Figures 1a and 1b

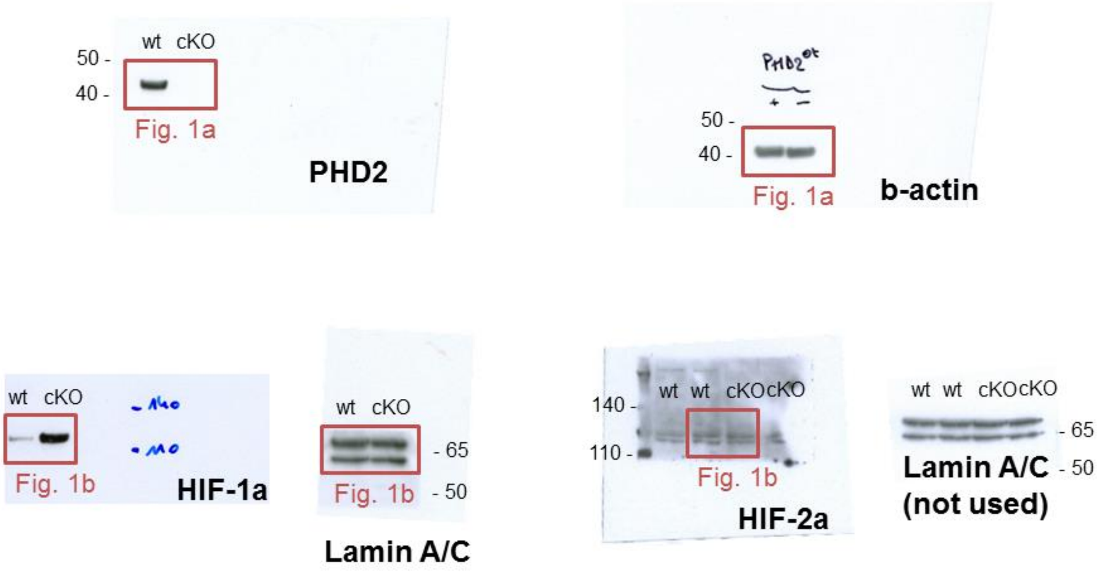

Figures 3b and c

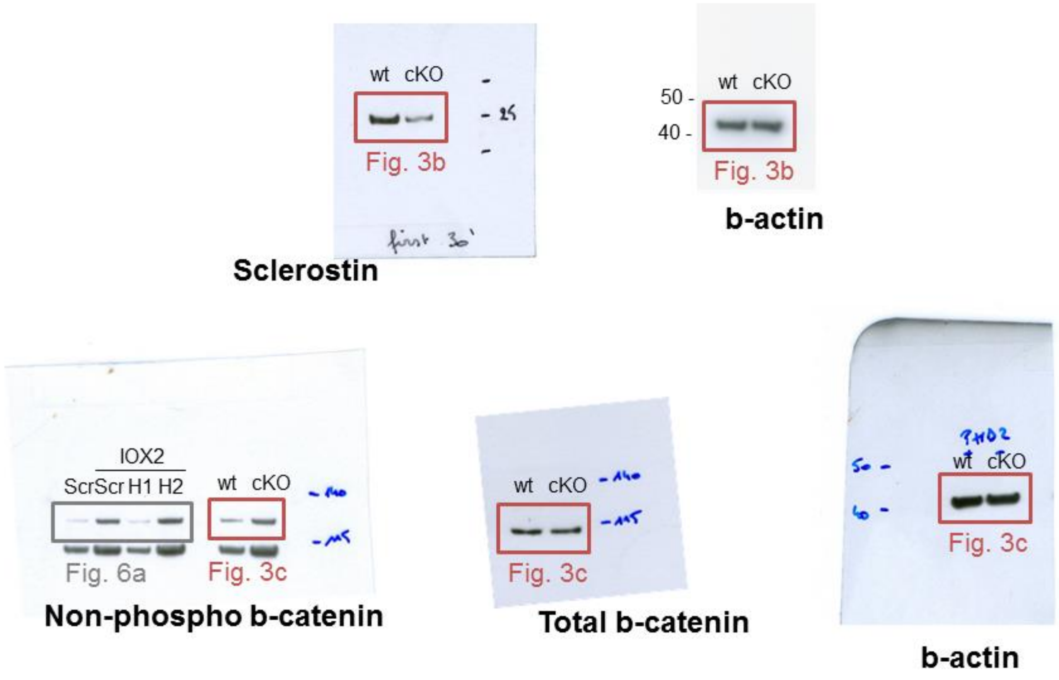

Figure 3e

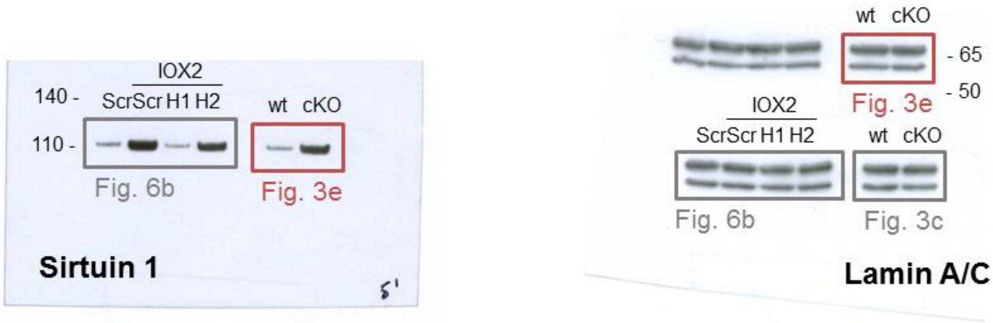

Figure 3f

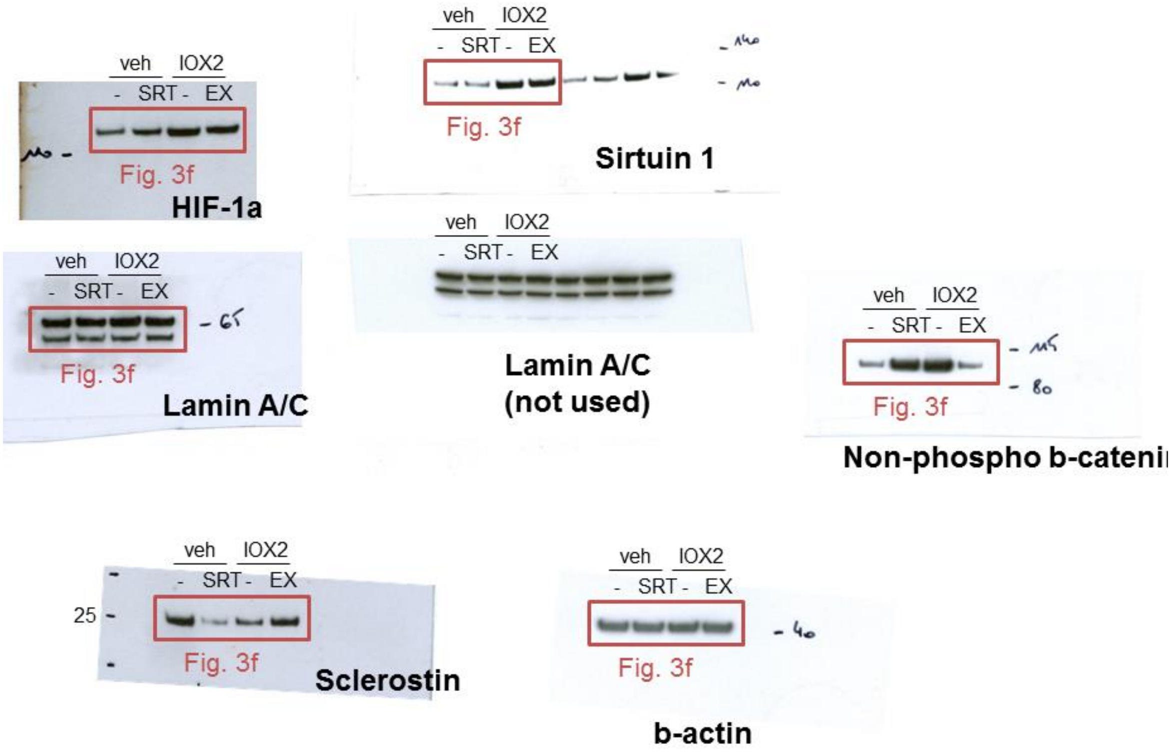

Figure 3g

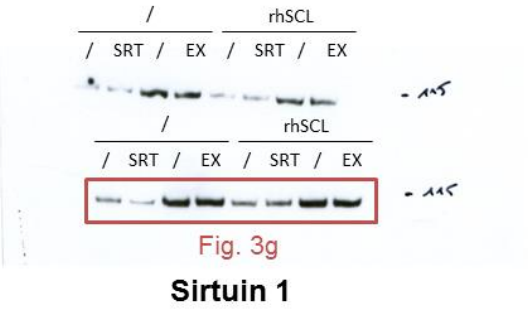

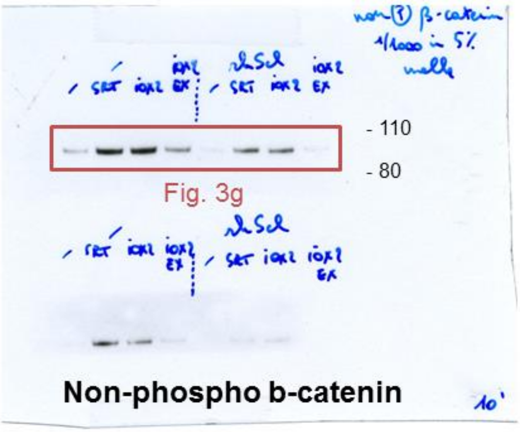

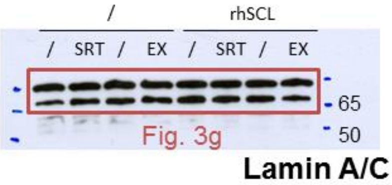

Figure 4d

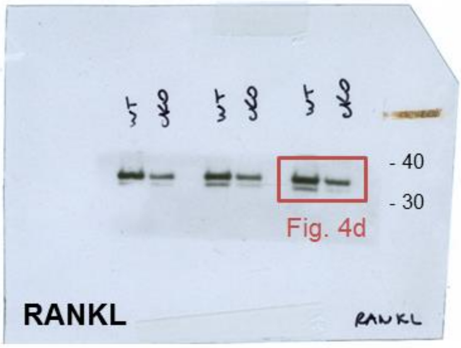

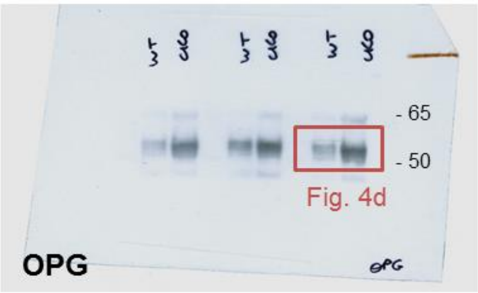

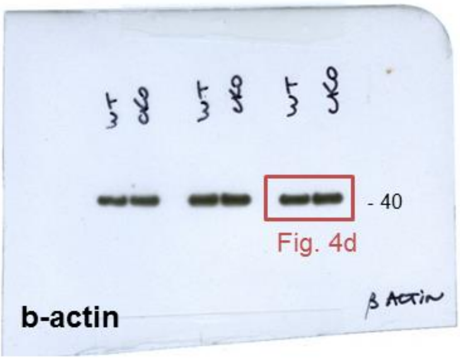

Figure 4e

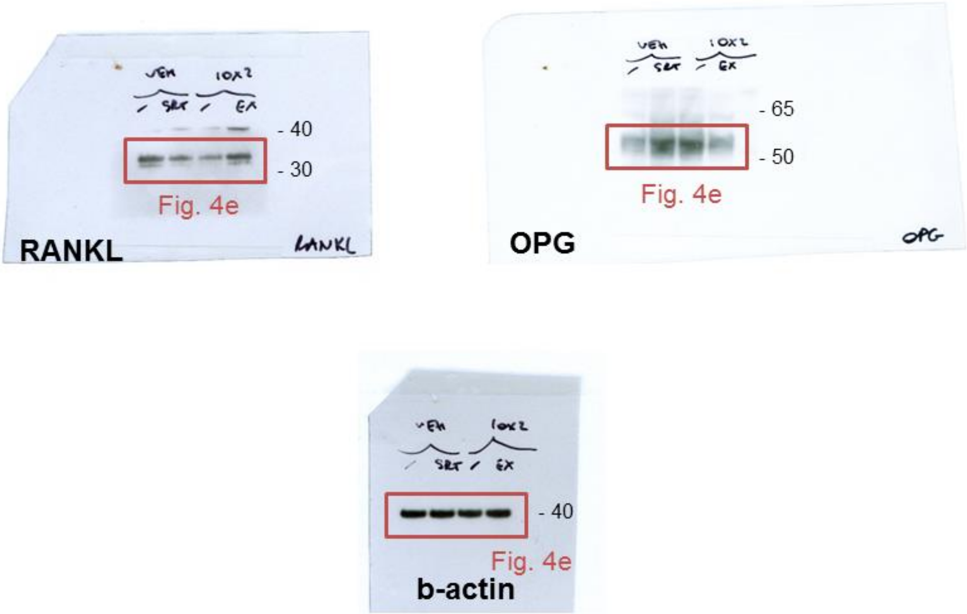

Figure 5j

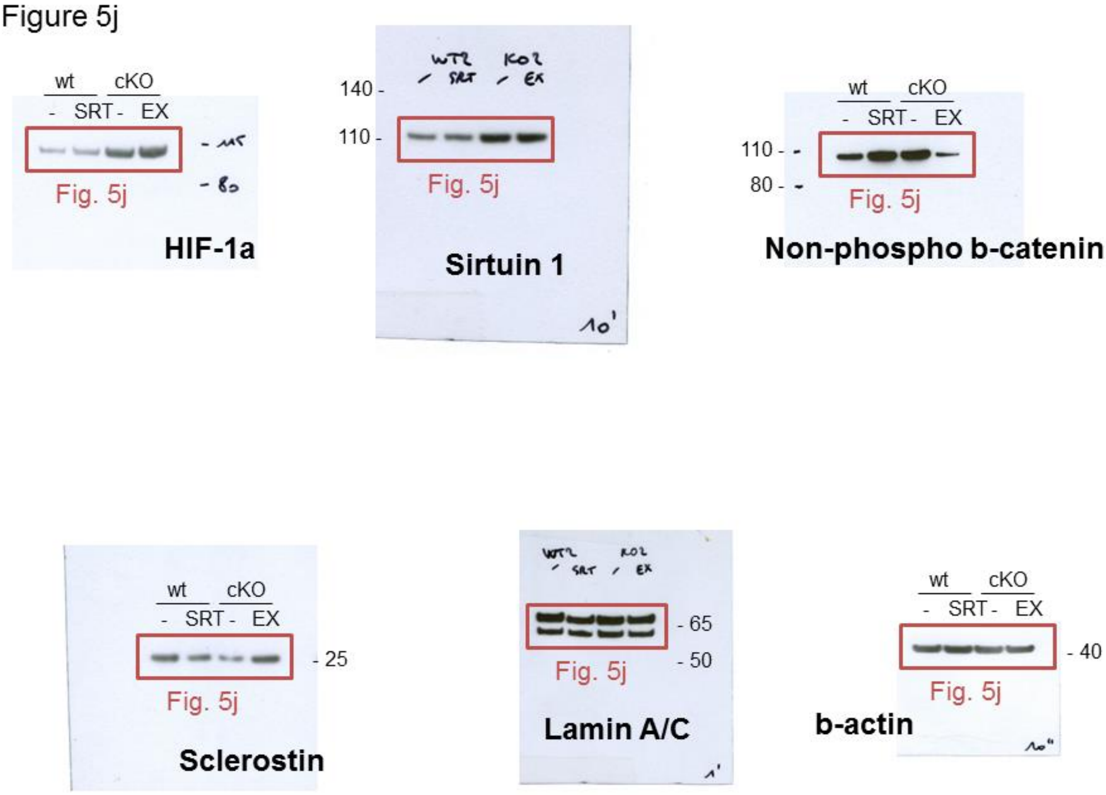

Figure 6a

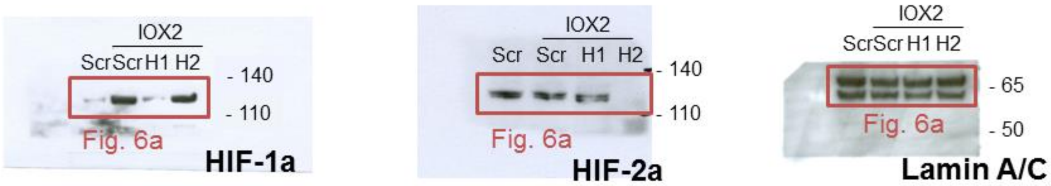

Figure 6b

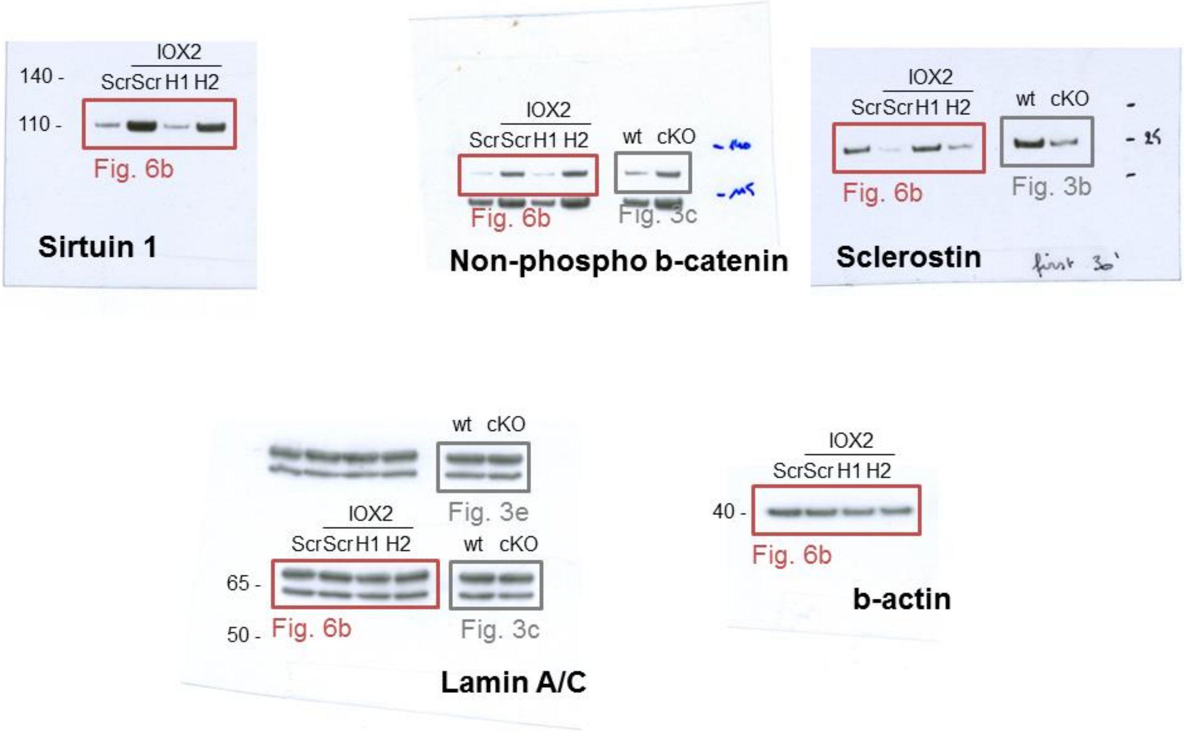

Figure 8j

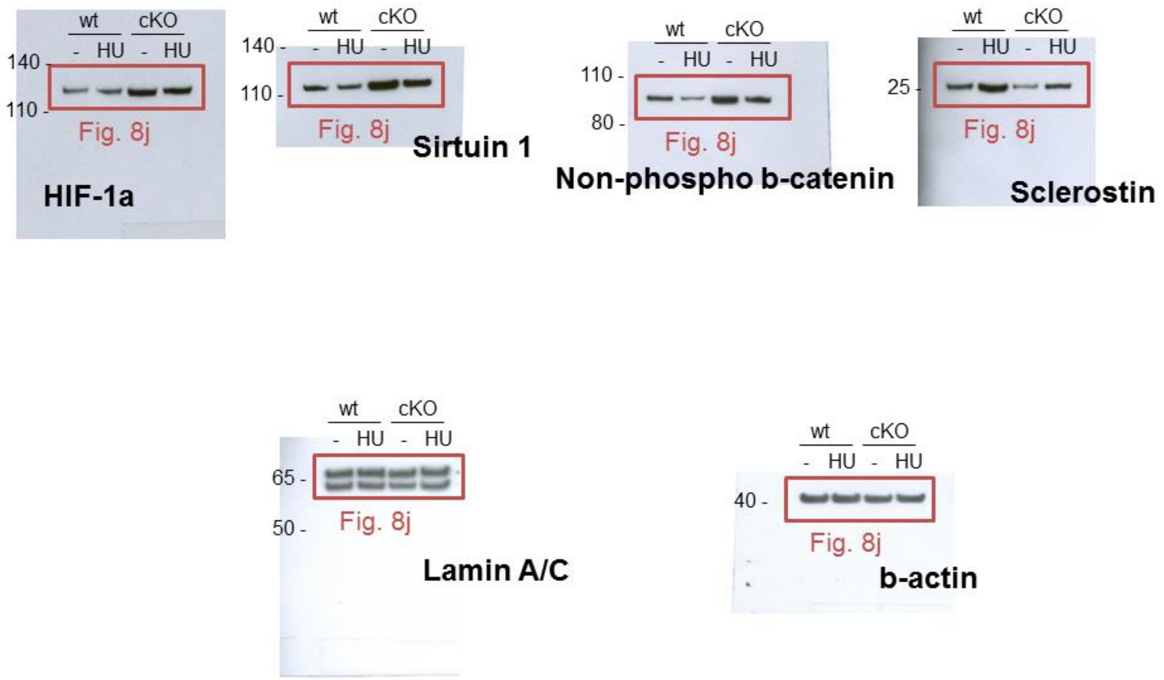

Figure 9j

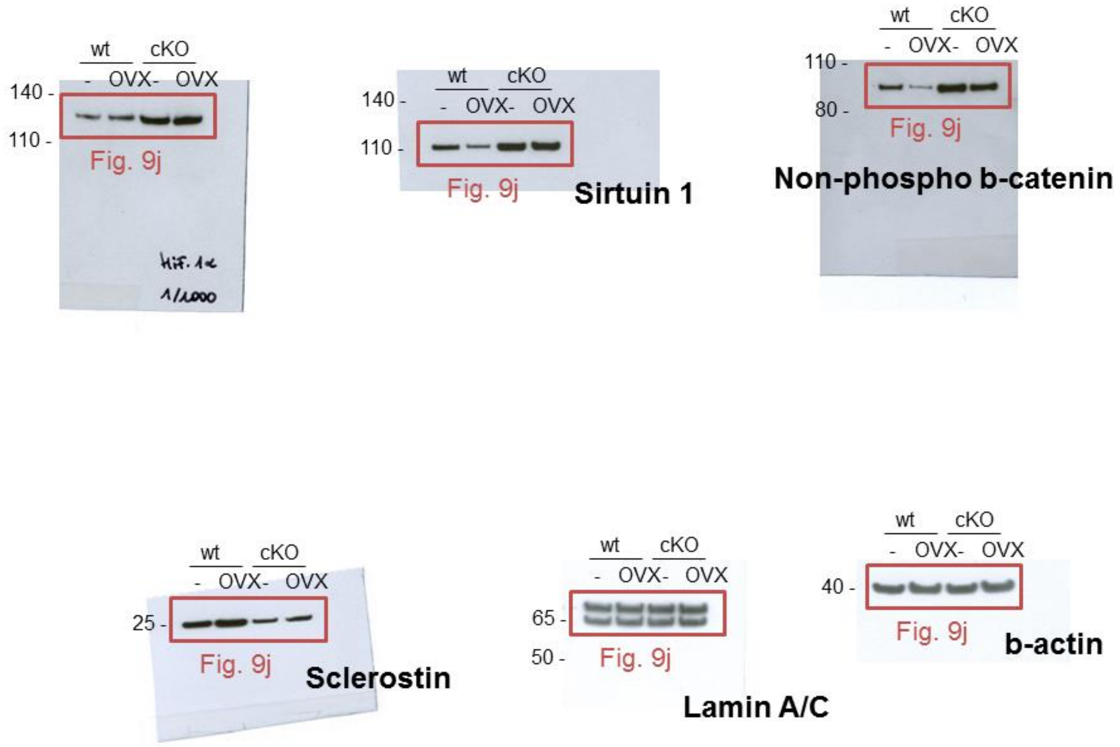

Supplementary Figure 1b

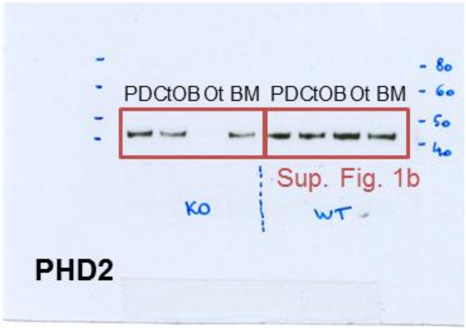

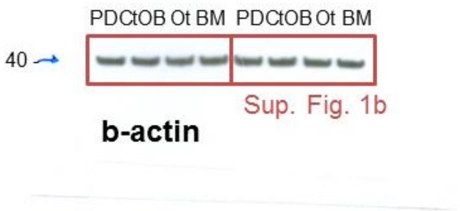

Supplementary Figure 6a

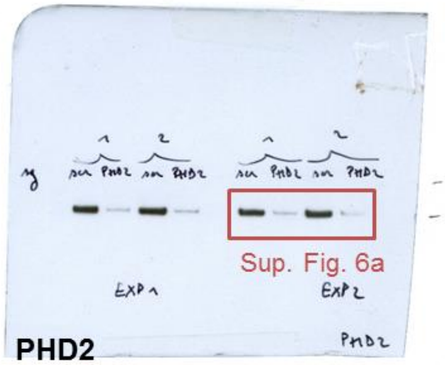

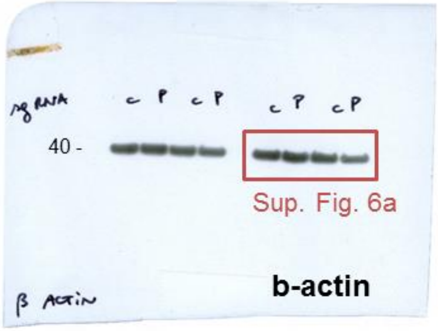

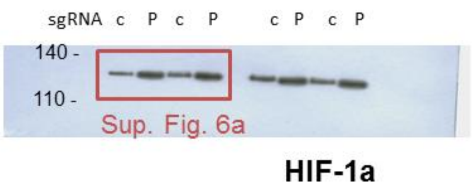

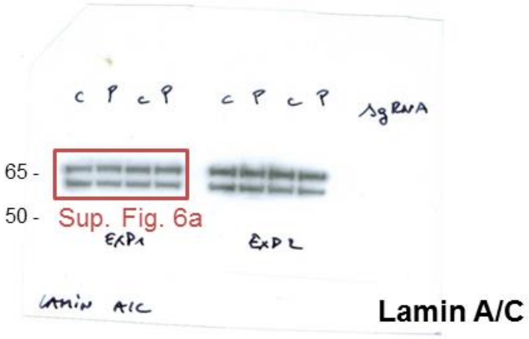

Supplementary Figure 6b

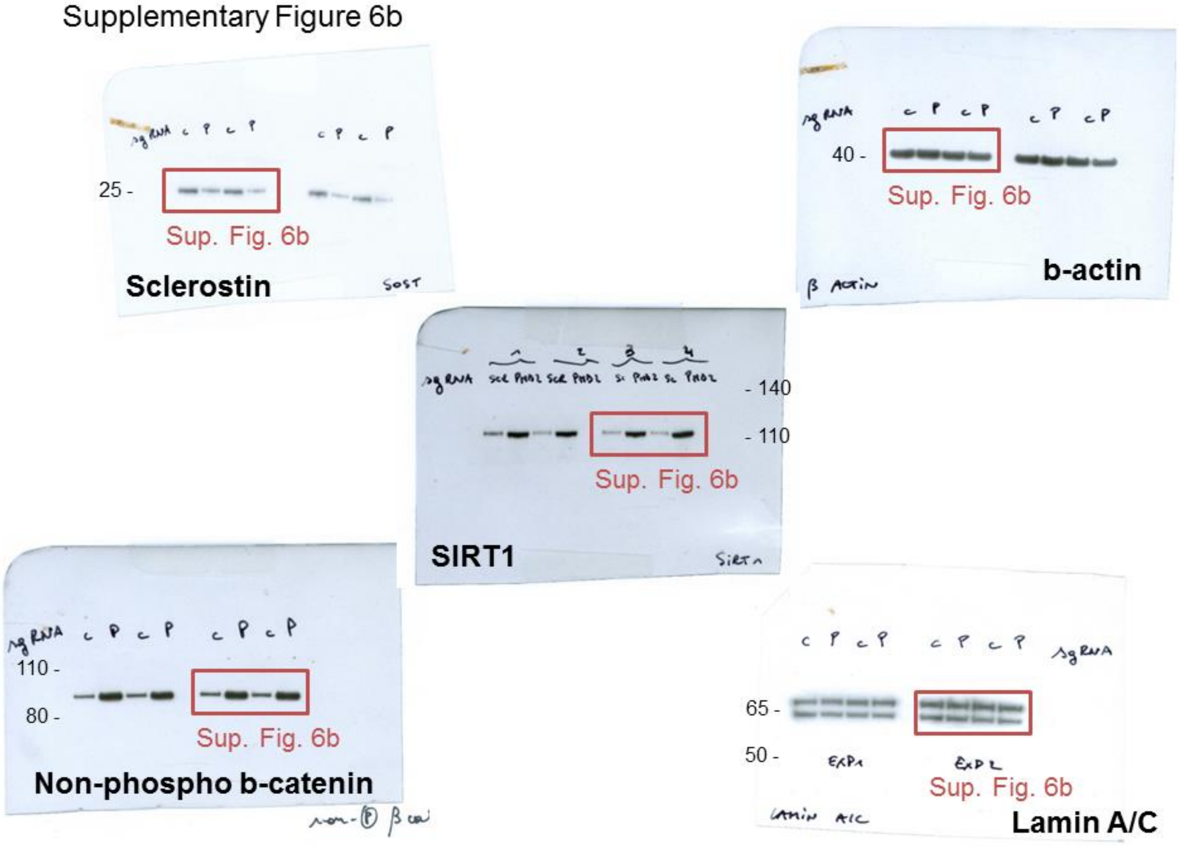

Supplementary Figure 6e

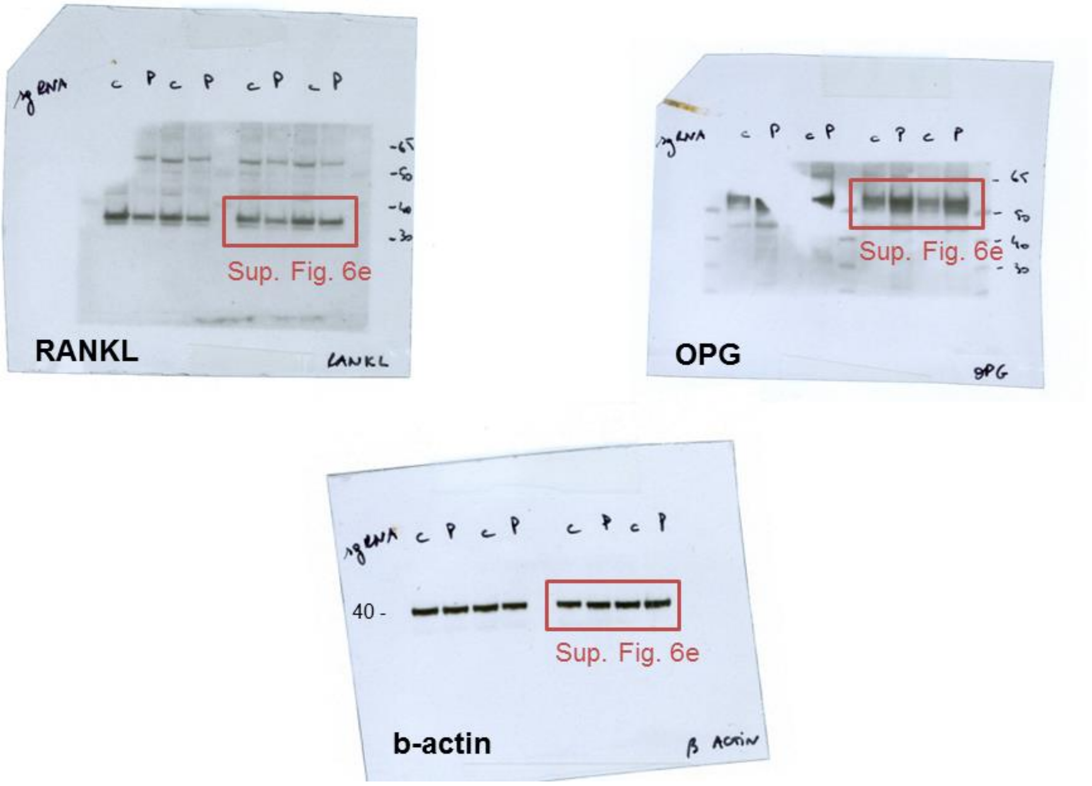

Supplementary Figure 8a

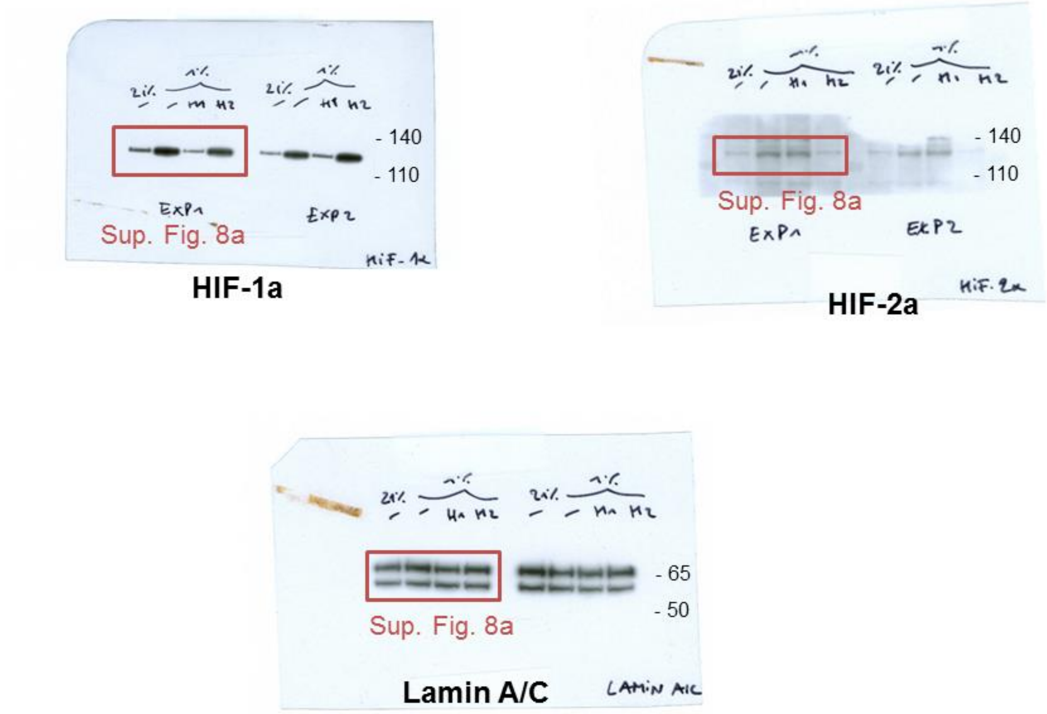

Supplementary Figure 8b

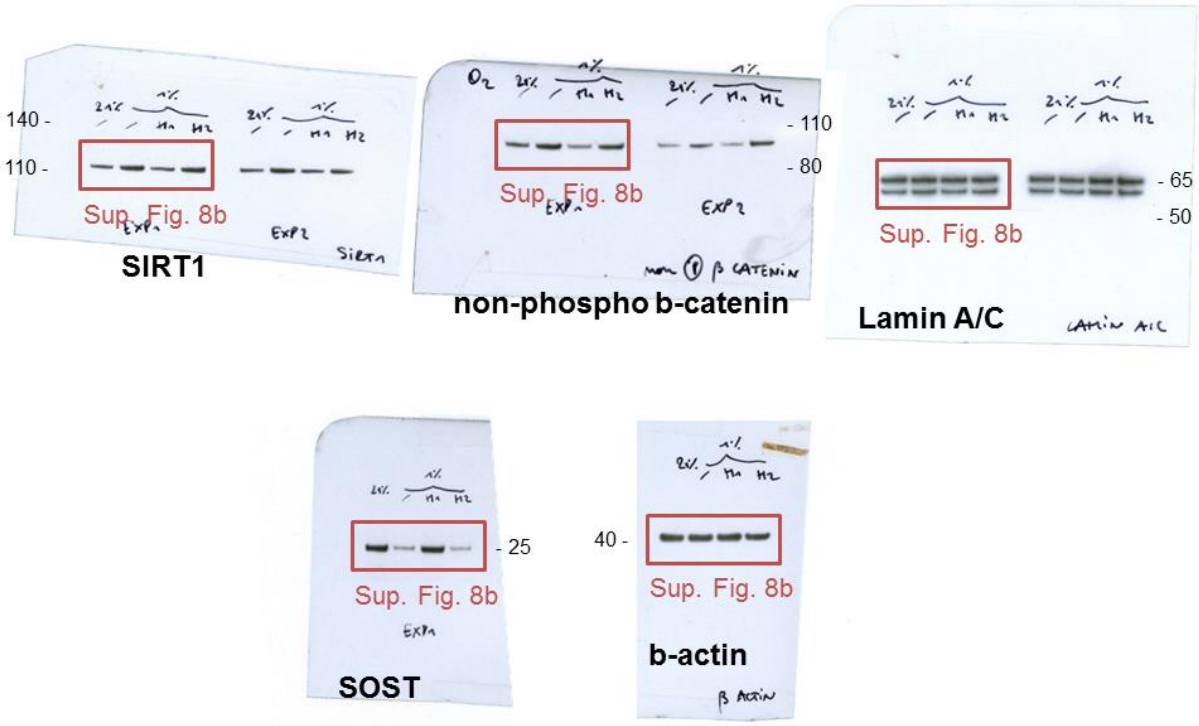

Supplementary Figure 9a

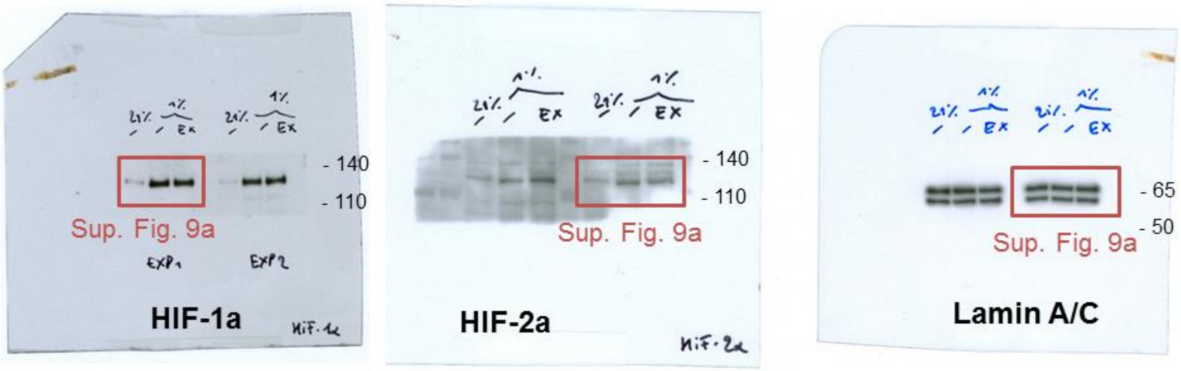

Supplementary Figure 9b

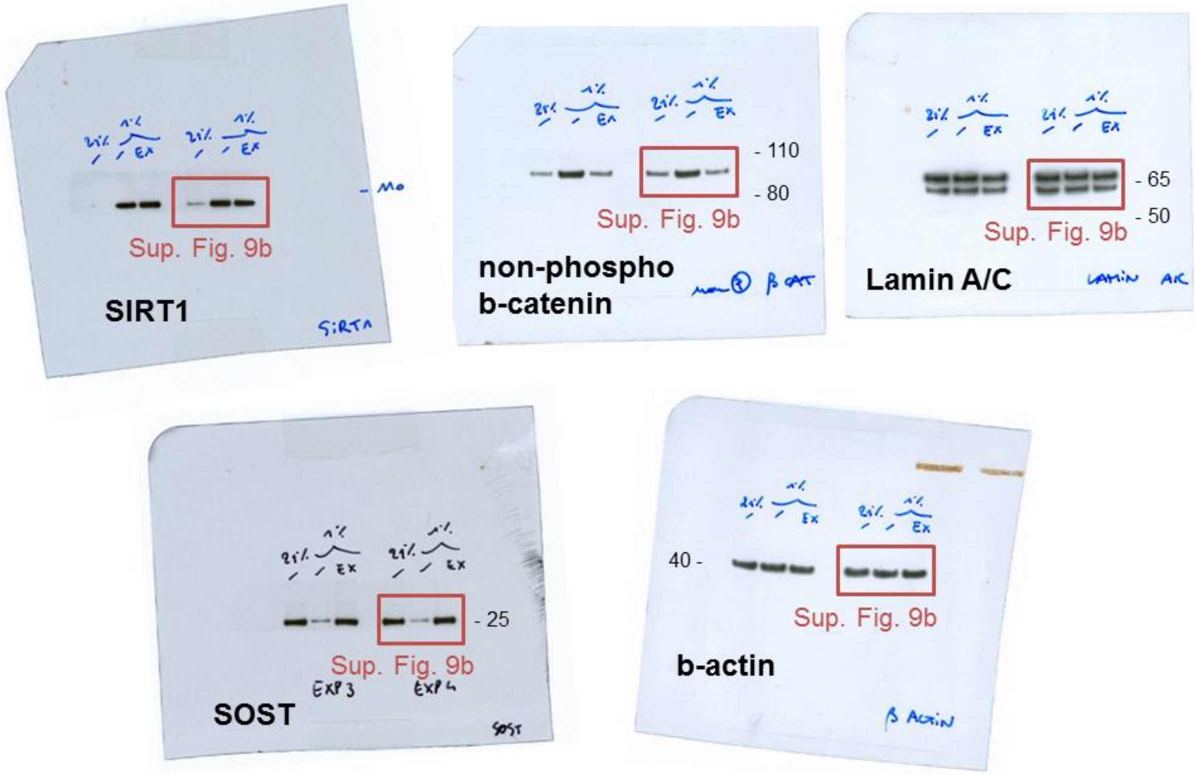

Supplementary Table 1. **Bone and gene expression analysis of hindlimb unloading (HU) model (related to figure 7)**

parameter	***Phd2^{ot+}***		***Phd2^{ot-}***		***genotype***	***treatment***	***genotype * treatment***
	grounded	*HU*	*grounded*	*HU*			
Body weight loss (%)	4.6±0.5	5.4 ± 0.7	4.6±0.6	5.7±0.3	N.S.	N.S.	N.S.
BV/TV (%)	10.6 ± 0.4	6.8 ± 0.9	22.3 ± 0.9	16.1 ± 0.9	p=0.0013	p=0.0022	p=0.0407
Ct.Th (mm)	0.18 ± 0.006	0.15 ± 0.004	0.20 ± 0.004	0.18 ± 0.004	p=0.0028	p=0.0025	p=0.0334
BFR ($\mu m^3/\mu m^2$/day)	0.86 ± 0.06	0.34 ± 0.07	1.50 ± 0.2	1.06 ± 0.09	p=0.0015	p=0.0481	p=0.0109
serum OCN (ng/ml)	44.8 ± 1.8	25.2 ± 2.6	112.8 ± 12.1	84.0 ± 2.7	p=0.0223	p=0.0420	N.S. (p=0.07)
Oc.S/B.S. (%)	18.4 ± 0.5	27.2 ± 2.0	13.2 ± 1.7	16.2 ± 2.5	p=0.0411	p=0.0299	p=0.0034
Rankl/*Opg* mRNA (fold change)	1.00 ± 0.11	1.82 ± 0.15	0.41 ± 0.07	0.59 ± 0.05	p=0.0111	p=0.0377	p=0.0032
Axin2 mRNA (fold change)	1.00 ± 0.09	0.67 ± 0.04	3.05 ± 0.36	2.16 ± 0.21	p=0.0206	p=0.0336	N.S.
Dkk-4 mRNA (fold change)	1.00 ± 0.25	0.62 ± 0.09	2.85 ± 0.25	2.32 ± 0.31	p=0.0301	p=0.0026	p=0.0461
Lef-1 mRNA (fold change)	1.00 ± 0.16	0.65 ± 0.09	6.52 ± 0.53	4.41 ± 0.42	p=0.0029	p=0.0387	N.S.
Tcf-1 mRNA (fold change)	1.00 ± 0.10	0.71 ± 0.06	2.52 ± 0.20	1.62 ± 0.11	p=0.0224	p=0.0178	N.S.

Two-way ANOVA analysis of genotype (*Phd2^{ot+}* vs. *Phd2^{ot-}*) * treatment (grounded vs. hindlimb unloading). Differences were considered to be significant when p<0.05, N.S. is not significant.

Supplementary Table 2. **Bone and gene expression analysis of ovariectomy (OVX) model (related to figure 8)**

parameter	***$Phd2^{ot+}$***		***$Phd2^{ot-}$***		***genotype***	***treatment***	***genotype * treatment***
	sham	*OVX*	*sham*	*OVX*			
Uterus weight (g)	235.9 ± 12.2	52.1 ± 4.4	232.7 ± 13.3	57.6 ± 3.2	N.S.	p=0.0032	N.S.
Body weight gain (%)	3.0 ± 0.7	4.6 ± 0.9	2.7 ± 0.5	4.0 ± 0.8	N.S.	N.S.	N.S.
BV/TV (%)	5.5 ± 1.0	2.5 ± 0.4	19.2 ± 1.6	11.9 ± 1.6	p=0.0143	p=0.0044	p=0.0345
Ct.Th (mm)	0.19 ± 0.002	0.17 ± 0.005	0.21 ± 0.006	0.20 ± 0.008	p=0.0387	p=0.0028	p=0.0136
BFR ($\mu m^3/\mu m^2$/day)	0.93 ± 0.08	1.09 ± 0.12	1.72 ± 0.06	1.55 ± 0.18	p=0.0035	p=0.0421	N.S.
serum OCN (ng/ml)	62.5 ± 8.2	78.0 ± 7.2	130.6 ± 5.2	148.5 ± 9.8	p=0.0079	p=0.0094	N.S.
Oc.S/B.S. (%)	22.5 ± 1.1	29.1 ± 1.7	17.3 ± 1.2	19.9 ± 0.7	p=0.0276	p=0.0013	p=0.0096
Rankl/*Opg* mRNA (fold change)	1.00 ± 0.08	6.32 ± 0.81	0.56 ± 0.09	1.36 ± 0.12	p=0.0055	p=0.0003	p=0.0034
Axin2 mRNA (fold change)	1.00 ± 0.10	0.84 ± 0.12	3.75 ± 0.42	3.56 ± 0.30	p=0.0167	p=0.0098	N.S.
Dkk-4 mRNA (fold change)	1.00 ± 0.09	0.75 ± 0.07	3.21 ± 0.31	2.52 ± 0.32	p=0.0209	p=0.0142	N.S. (p=0.05)
Lef-1 mRNA (fold change)	1.00 ± 0.12	0.69 ± 0.09	5.36 ± 0.42	4.32 ± 0.57	p=0.0065	p=0.0052	N.S.
Tcf-1 mRNA (fold change)	1.00 ± 0.08	0.57 ± 0.06	2.35 ± 0.24	2.62 ± 0.21	p=0.0354	p=0.0116	p=0.0408

Two-way ANOVA analysis of genotype (*$Phd2^{ot+}$* vs. *$Phd2^{ot-}$*) * treatment (sham vs. OVX). Differences were considered to be significant when $p<0.05$, N.S. is not significant.

Supplementary Table 3. **Oligonucleotide sequences used for qRT-PCR analysis.**

Gene		***Oligonucleotide sequence***
Axin2		Commercial: Mm.PT.58.8726473
Col1	Forward<br>Reverse<br>Probe	5’-TGTCCCAACCCCCAAAGAC-3’<br>5’-CCCTCGACTCCTACATCTTCTGA-3’<br>5’-ACGTATTCTTCCGGGCAGAAAGCACA-3’
Dkk-4		Commercial: Mm.PT.58.9461861
Dmp1	Forward<br>Reverse<br>Probe	5’-TGTCATTCTCCTTGTGTTCCTTTG-3’<br>5’-AATCACCCGTCCTCTCTTCAGA-3’<br>5’-CTGTCCTGTGCTCTCCCAGTTGCCA-3’
Hprt	Forward<br>Reverse<br>Probe	5’-TTATCAGACTGAAGAGCTACTGTAATGATC-3’<br>5’-TTACCAGTGTCAATTATATCTTCAACAATC-3’<br>5’-TGAGAGATCATCTCCACCAATAACTTTTATGTCCC-3’
Lef-1		Commercial: Mm.PT.58.33138498
Ocn	Forward<br>Reverse<br>Probe	5’-GGCCCTGAGTCTGACAAAGC-3’<br>5’-GCTCGTCACAAGCAGGGTTAA-3’<br>5’-ACAGACTCCGGCGCTACCTTGGAGC-3’
Opg	Forward<br>Reverse<br>Probe	5’-GAAGGGCGTTACCTGGAGATC-3’<br>5’-CTGAATTAGCAGGAGGCCAAAT-3’<br>5’-TCACCTGAGAAGAACCCATCTGGACATTTT-3’
Phd2	Forward<br>Reverse<br>Probe	5’-CTGGGCAACTACAGGATAA-3’<br>5’-CATAGCCTGTTCCGTTGCCT-3’<br>5’-ACGAAAGCCATGGTTGCTTGT-3’
Phex	Forward<br>Reverse<br>Probe	5’-GGATATTAGGTGCCGAGAGGAA-3’<br>5’-TACCCTTGAGAATTCTAGCCATCTATATT-3’<br>5’-TTATTCCAGAATTCCAAACCTCAGCAGGC-3’
Plgf	Forward<br>Reverse<br>Probe	5’-TTCAGTCCGTCCTGTGTCCTT-3’<br>5’-GCACACAGTGCAGACCTTCA-3’<br>5’-ACCACAGCAGCCACTACAGCGACTCA-3’
Rankl	Forward<br>Reverse<br>Probe	5’-CATTTGCACACCTCACCATCA-3’<br>5’-TTGCTTAACGTCATGTTAGAGATCTTG-3’<br>5’-TCGGGTTCCCATAAAGTCACTCTGTCCTCTT-3’
Runx2	Forward<br>Reverse<br>Probe	5’-TACCAGCCACCGAGACCAA-3’<br>5’-AGAGGCTGTTTGACGCCATAG-3’<br>5’-CTTGTGCCCTCTGTTGTAAATACTGCTTGCA-3’
Sirt1		Commercial: Mm.PT.58.17360475
Sirt2		Commercial: Mm.PT.56a.31622012
Sirt3		Commercial: Mm.PT.58.8392007
Sirt4		Commercial: Mm.PT.58.9278586
Sirt5		Commercial: Mm.PT.58.31692891
Sirt6		Commercial: Mm.PT.56a.33600211
Sirt7		Commercial: Mm.PT.58.29943064
Sost	Forward<br>Reverse<br>Probe	5’-CCTGAGAACAACCAGACCATGA-3’<br>5’-TGGGCCGTCTGTCAGGAA-3’<br>5’-TCCGAGTACAGCTGCCGCGAGC-3’
Tcf-1		Commercial: Mm.PT.58.43503487

Vegf	Forward<br>Reverse<br>Probe	5’-AGTCCCATGAAGTGATCAAGTTCA-3’<br>5’-ATCCGCATGATCTGCATGG-3’<br>5’-TGCCCACGTCAGAGAGCAACATCAC-3’

Supplementary Table 4. **Oligonucleotide sequences used for ChIP-qPCR analysis.**

	Primer sequence	
	forward	*reverse*
Positive control (SIRT1 ChIP)	5'-GACGACTTGAAAAATGACGAAATC-3'	5'-CATATTCCAGGTCCTTCAGTGTGC-3'
Negative control (SIRT1 ChIP)	5'-AGAGTCGTACCTCCAAGGGC-3'	5'-CTGGATCCTCTGGACCTGG-3'
Positive control (H3K9Ac ChIP)	5'-CTTTGAGGCTACAGGGTGGA-3'	5'-GAGAAGCCTTCCCCATCTTC-3'
Negative control (H3K9Ac ChIP)	5'-GGTCAGTGGCAAGGAACATT-3'	5'-TTGTTCCTTTGATGGGCAAT-3'
Sost promoter region 1 (-998-1115)	5'-GGAGACCTGAGGAGGTGTCTT-3'	5'-GTGGATTCTGGGGGCTTACT-3'
Sost promoter region 2 (-761-844)	5'-TTCAAAACCAGTGGGAGAGG-3'	5'-ACCATGAGTTCCCTGGCTAA-3'
